# PatientEase—Domain-Aware RAG for Rehabilitation Instruction Simplification

**DOI:** 10.3390/bioengineering12111204

**Published:** 2025-11-03

**Authors:** Rashid Nasimov, Akmalbek Abdusalomov, Charos Khidirova, Khosiyat Temirova, Alpamis Kutlimuratov, Shakhnoza Sadikova, Wonjun Jeong, Hyoungsun Choi, Taeg Keun Whangbo

**Affiliations:** 1 Department of Computer Engineering, Gachon University Sujeong-Gu, Seongnam-si 13120, Gyeonggi-Do, Republic of Korea; rashid.nasimov@tsue.uz (R.N.); tp04045@gachon.ac.kr (W.J.); hschoi@gachon.ac.kr (H.C.); 2 Department of Computer Systems/Artificial Intelligence, Tashkent University of Information Technologies Named After Muhammad Al-Khwarizmi, Tashkent 100200, Uzbekistan; akmaljon@gachon.ac.kr (A.A.); khidirova@tuit.uz (C.K.); xosiyattemirova6033@gmail.com (K.T.); 3 Department of Artificial Intelligence, Tashkent State University of Economics, Tashkent 100066, Uzbekistan; 4 Department of Information Processing and Management Systems, Tashkent State Technical University, Tashkent 100095, Uzbekistan; sshakhnoza@yandex.ru; 5 Department of International Scientific Journals and Rankings, Alfraganus University, Yukori Karakamish Street 2a, Tashkent 100190, Uzbekistan; 6 Department of Applied Informatics, Kimyo International University in Tashkent, Tashkent 100121, Uzbekistan; kutlimuratov.aj@kiut.uz

**Keywords:** medical text simplification, retrieval-augmented generation (RAG), multi-agent coordination, domain adaptation, reinforcement learning from human feedback (RLHF), clinical natural language processing (NLP), patient-centered healthcare communication, rehabilitation informatics

## Abstract

Background: Rehabilitation depends on using instructional materials, which many patients find difficult to understand; thus, their adherence to the safety and care may be affected. Text simplification systems used, in general, do not usually focus on procedure-oriented guidance or the degree of personalization required in rehabilitation settings. Methods: We present PatientEase, a domain-aware retrieval-augmented generation framework that changes rehabilitation instructions to simple words without changing the clinical meaning. PatientEase incorporates two complementary retrievers that is a corpus retriever that is tuned for rehabilitation and a user-aligned retriever that is conditioned on patient profiles, together with a role-structured, multi-agent rewriting pipeline; outputs can be further refined by using reinforcement learning from human feedback with a composite reward for readability, factuality, and clinician-preferred structure. Results: The latter was quite comprehensively compared in four benchmark tests against baselines, wherein SARI, FKGL, BERTScore, and MedEntail indices are employed, as well as clinician-patient assessments. PatientEase achieves 52.7 SARI and 92.1% factual entailment, and receives the highest fluency and simplicity ratings; ablations also underline each module’s role. Conclusions: PatientEase paves the road for safer, patient-centered communication in rehabilitation and lays the groundwork for trustworthy clinical dialogue systems.

## 1. Introduction

Communication in healthcare serves as a cornerstone for engaging patients, ensuring treatment adherence, and achieving positive health outcomes [1]. This is even more vital within the rehabilitation context, where patients undergo prolonged therapy spanning across physical, cognitive, and speech rehabilitation [2]. However, much of the supplementary written material that guides patients through these processes, for example, discharge summaries and therapy instructions, is composed of complex medical jargon, acronyms, and stratified syntax [3]. These verbal strokes are especially harmful for older adults and those with cognitive decline, low health literacy, and limited education, all of whom make up a considerable proportion of the rehabilitation population [4]. Recent literature demonstrates a troubling gap between the medical reading materials provided and the reading skills of the intended audience [5]. Despite this, analyses show that most clinical instructions are composed at a tenth-grade level or higher, deepening the chasm of understanding, particularly among underserved and multilingual populations [6]. Within rehabilitation settings, where practitioner autonomy and habitual performance drive patient progress, the detrimental consequences of miscommunication include the full range of non-compliance, prolonged recovery, and unnecessary readmissions [7].

The development of large language models (LLMs) has provided new opportunities for the automatic simplification of complex texts [8]. Models based on transformers like T5, BART, and GPT-4 have been powerful in performing tasks like machine translation and text rewriting or transforming texts into summaries in a more sophisticated and intelligent manner [9]. Yet, there is still a biomedical gap between LLMs and the application for medical term simplification due to two principal shortcomings. Generic LLMs do not have any simplification domain cognizance, which would either result in paraphrasing or omitting medically pertinent details. Domain adaptation to user-specific parameters such as language level, cognitive ability, and familiarity with the topic, especially in rehabilitation contexts, is crucial. Unchecked simplification can obscure meaning or lead to hallucinations, leading to severe risks for a patient’s well-being. To fill these gaps, we introduce PatientEase: A new Domain-Aware RAG framework tailored for medical term simplification within rehabilitation contexts. Our method incorporates three fundamental principles. Simplification stays clinically relevant and lexically accessible with the implementation of a dual retrieval structure that merges a domain-specific retriever trained on rehabilitation materials with a user-aligned retriever tuned to the patient’s vocabulary and comprehension profile. Second, PatientEase defines a multi-agent simplification loop comprising transformer-based agents with specific roles for domain validation, lay translation, simplification of syntax, and redundancy trimming. This modular decomposition emulates workflows of trained human specialists and provides robust mitigating partitioning to structural damages for simplification. Third, the system is fine-tuned using Reinforcement Learning with Human Feedback (RLHF) with a composite signal encapsulating readability, semantic accuracy, and factual precision from expert reviewers and evaluator avatars from patient personas posing as patients as the reward signal.

The framework introduces three key innovations:

- A dual-retrieval engine combining a domain-specific retriever and a user-aligned retriever.
- A multi-agent simplification loop that distributes rewriting tasks among specialized transformer agents responsible for lay translation, domain validation, and syntactic restructuring.
- A reinforcement learning optimization phase guided by human and simulated clinical feedback, optimizing for readability, factual accuracy, and clinician-preferred style.

PatientEase is the first framework that jointly integrates domain awareness, user personalization, and RLHF optimization for safe medical simplification in rehabilitation. By aligning text complexity with patient literacy while preserving the original medical meaning, the system contributes to trustworthy AI for patient-centered communication, a critical advancement for improving treatment adherence and clinical safety in modern rehabilitation practice.

## 2. Related Works

Improving healthcare communication has always aimed to parse complex medical texts into simpler terms to improve text accessibility. In rehabilitation therapy—physical therapy, speech recuperation, and cognitive retraining—effective communication is important, but in this situation, it is vital. Efforts toward the simplification of medical texts have, however, centered on general biomedical frameworks or academic abstracts, paying little attention to the procedural and instructional rehabilitation texts’ frameworks. Recent developments of transformer-based language models, BART [10], T5 [9], and GPT-4 [11], have greatly enhanced the automation of text simplification. These models have performed impressively in translation, summary generation, and rewriting processes [12,13]. Nonetheless, their use in medical simplification—more so in rehabilitation contexts—remains restricted because of three major issues: lack of tailoring at the domain level, personalization at the user level, and fragility in critical safety situations [14,15]. To situate our contribution, we focus on four intersecting areas for the design of PatientEase: biomedical text simplification, RAG, domain adaptation of LLMs, and RLHF.

The early works of biomedical text simplification focused on a combination of rule-based approaches and substitution of terms based on frequency [16,17]. Although these approaches attempted to replace terms with simpler equivalents, they were unable to restructure complex syntax or maintain contextual semantics during the simplification process [18]. More recent works have utilized encoder–decoder models such as BART and T5 for end-to-end simplification. As highlighted by other studies, such models have proven capable of transforming biomedical abstracts into summary form [19]. However, these models are adapted to formal scientific texts and perform poorly on rehabilitation texts that require clarity and function-focused understandability from the performing discourse [20]. Generative systems suffer from hallucinations and other issues with factual precision. RAG has surfaced as a successful approach to enhancing precision in such systems. RAG is a method first presented by Lewis et al. [21] that augments contextual inputs for generation with outside documents. In the biomedical domain, extensions such as BioRAG [22] and MedSyn [23] have shown higher effectiveness in ICD code generation, answering questions, and document retrieval [24]. But these models focus on inference and QA, leading to a lack of attention given to rewriting at the sentence level. Moreover, they do not consider variability of understanding, which is very important in rehabilitation, where the cognitive and educational profiles of patients differ is a wide range [25,26]. The domain adaptation of LLMs led to the development of models pre-trained on biomedical texts such as SciFive [27], BioBART, and PubMedGPT [28]. These models perform well on domain-specific summarization and named entity recognition. Still, on summarization, their outputs are laden with, and often incorporate, field-specific terminology that is inaccessible to non-experts. BioGPT [29] is one such example widely known to generate very accurate outputs for medical texts, but fails to rephrase words and phrases in a way that patients understand. Although control tokens and stylistic prompts [30] have been applied for improving readability, these approaches lower the precision of the presented information or require extreme tuning in a specific domain. Reinforcement Learning with Human Feedback (RLHF) is known to be a better approach for controlling a model’s output to align with human expectations. An example is GPT-4 that uses OpenAI’s technology, where dialogue quality and safety of facts have been improved through the use of RLHF. GPT-4 is well known for dialogue quality and safety of facts being improved through RLHF [31]. Complementarily, attention-centric and hybrid architectures in adjacent domains show how multi-domain dynamic attention can enhance both performance and interpretability, motivating our multi-agent, retrieval-grounded design [32].

In recent years, research on RAG and reinforcement-driven text simplification has accelerated, particularly within clinical and patient-education domains. Several new studies have substantially advanced the methodological landscape and are directly aligned with the objectives of the PatientEase framework. Rahman et al. introduced an annotated corpus for digestive cancer education and demonstrated how reinforcement learning strategies could be used to simplify medical texts while maintaining semantic integrity and factual grounding [33]. This study provides the first large-scale resource combining reinforcement-based optimization with domain-controlled health literacy. Similarly, Ayre et al. evaluated an Online Plain Language Tool that improves the readability and trustworthiness of patient information on public health websites, demonstrating the practical importance of readability-focused AI interventions [34]. Recent reviews have emphasized the expanding role of RAG in healthcare contexts. Gaber et al. presented a systematic review of retrieval-augmented generation for medical applications, mapping its use in clinical question answering, report generation, and patient education [35]. Complementary overviews by Gargari et al. provided concise and comprehensive analyses of RAG-driven medical NLP pipelines, underscoring their potential to improve both factual reliability and interpretability in AI-assisted healthcare communication [36]. The growing consensus across these works affirms that domain-aware RAG systems can bridge the gap between linguistic fluency and factual precision, provided they incorporate user-centered adaptation and iterative feedback. Building upon these insights, PatientEase introduces a dual-retrieval and multi-agent simplification pipeline specifically tailored for rehabilitation instructions—an area not yet addressed in prior RAG research.

In medical NLP, some researchers used RLHF to improve the readability and clinical safety of texts with composite functions involving entailment, fluency, and comprehension. Nonetheless, applying these techniques to the automation of explanatory texts, especially those that use command or action-oriented language on tasks in rehabilitation, has yet to be adequately addressed. Furthermore, no other framework has been proposed that systematically combines RLHF with retrieval and agent-based generation in this specific context. Although all four areas we discussed inform critical foundational concerns, none of them individually fulfills the two requirements: domain-aware and user-personalized simplification in rehabilitation. To fill this gap, we present PatientEase, a new framework that combines retrieval-augmented generation with domain-aware retrieval, user-aligned adaptation, multi-agent simplification, and reinforcement learning with human feedback optimization into a single system. This closes the methodological void between generic NLP research and patient dialogue in rehabilitation contexts.

## 3. Materials and Methods

**Figure 1:**
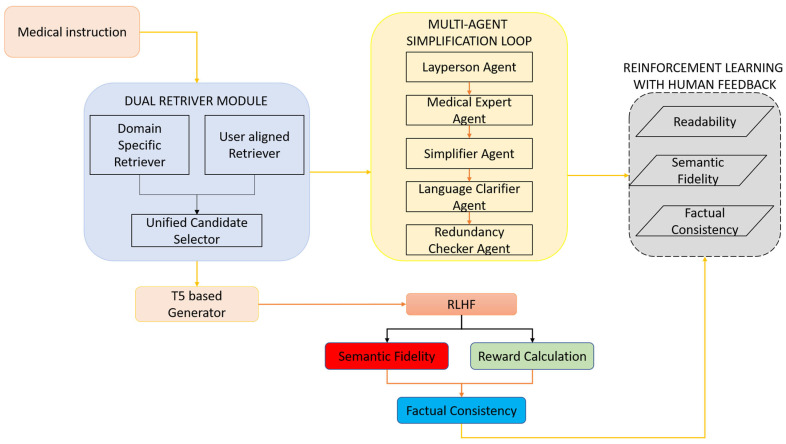
Overview of the PatientEase Architecture. The proposed PatientEase framework consists of three primary modules: (1) a Dual Retriever Module combining a domain-specific retriever and a user-aligned retriever to provide contextually and cognitively relevant reference material; (2) a Multi-Agent Simplification Loop that distributes simplification tasks across specialized agents, including layperson detection, medical validation, syntactic simplification, lexical clarification, and redundancy removal; and (3) an RLHF Optimization Phase that refines output quality through reinforcement learning using composite reward signals based on readability, semantic fidelity, and factual consistency. A T5-based generator acts as the backbone of the system, integrating retrieval-based evidence and agent-guided edits.

Existing methods for medical text simplification often struggle with three persistent shortcomings: they pay insufficient attention to the nuances of clinical language, they fail to align explanations with the unique backgrounds of individual users, and they do not guarantee patient safety when information is resized or rewritten. This paper introduces PatientEase, a Retrieval-Augmented Generation framework specifically engineered for rehabilitation discourse. The underlying design features three interlocking modules: a dual-retrieval engine that mines both closed-domain knowledge sources and user-comprehension profiles, a multi-agent rewriting loop that parcels rewriting tasks among specialized transformer instantiations, and a final reinforcement-learning pass shaped by human judgments about fluency, semantic integrity, and factual correctness. Each of these building blocks-narrated here in turn-along with the curated datasets, benchmarking protocol, and experimental yardsticks, situates PatientEase against current state-of-the-art baselines Figure 1.

### 3.1. Dual Retrieval Module

**Figure 2:**
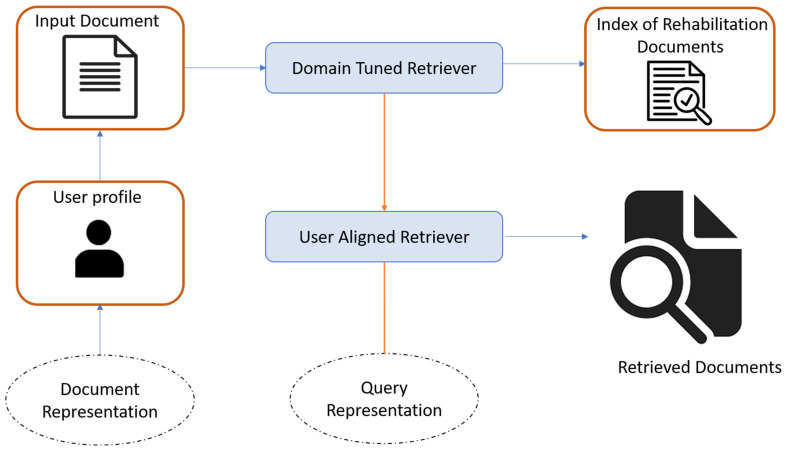
The Dual Retrieval Module of PatientEase. This architecture incorporates a Domain-Tuned Retriever, trained on medical and rehabilitation texts, and a User-Aligned Retriever, guided by individual patient profiles. Together, they generate a contextually relevant and patient-personalized evidence pool for downstream simplification.

The use of two retrievers in PatientEase is grounded in complementary design objectives. The domain retriever ensures that all retrieved candidates preserve the exact semantics and specialized terminology of rehabilitation medicine, maintaining clinical validity and factual alignment. Meanwhile, the user-aligned retriever adapts retrieval selection to the reader’s cognitive and literacy profile, measured through dynamic control vectors encoding readability metrics. While a single unified retriever was initially tested, its representations failed to balance these competing objectives. The unified retriever tended to overgeneralize—sacrificing domain precision for simplicity—resulting in terminological drift and loss of critical safety cues in rehabilitation contexts. In contrast, the dual-retrieval setup allows the system to blend medically grounded content with personalized linguistic adaptation, creating an evidence pool that simultaneously respects factuality and accessibility. The PatientEase framework utilizes a dual retrieval approach to facilitate domain-informed and user-aligned simplification generation. This module resolves two of the most important issues: maintaining the rehabilitation medicine domains’ semantic fidelity of the retrieved content, and adapting the simplification retrievals to the blended cognition and language profiles of various patient populations. $x\in R^{d}$ encodes the embedding of the S sentence, where S is an input sentence composed of intricate medical words and phrases. About the system that has been designed, one of the modules, which is aimed at retrieving the simplification candidates, is tasked with finding a set of candidates $\left\{C_{1},C_{2},\ldots ,C_{k}\right\}$ which have the highest relevancy alongside user alignments. This will be performed while treating them as an augmentation to aid the generator. To accomplish this, we set up two separate retrievers. The first one is a domain-specific retriever such as the one that focuses on finding the clinically accurate medical term simplifications based on the dependence of their clinical relevance. For a given query embedding x, the retriever selects k items from $D_{domain}$ such that:

(1) $${Score}_{domain}\left(x,c_{i}\right)=cos\left(x,f_{\theta }\left(c_{i}\right)\right)$$

where $f_{\theta }$ is a cos (.,.) similarity BERT-based encoder trained with triplet loss on rehabilitation-aligned sentence pairs. The domain retriever was fine-tuned on the Med-EASI and SimpleDC datasets, as well as on a set of manually curated rehabilitation discharge summaries, thus infusing medical knowledge into a high-dimensional space where semantic similarity as well as terminological precision is preserved Figure 2.

At the same moment, the audience-sensitive retriever zeroes in on pulling up simplification samples that respect the linguistic habits and cognitive load of its particular readership. To anchor that focus, the original query *x* is blended with a control embedding *u* from the d-dimensional space $R^{d}$, the vector u encodes user-driven cues such as projected reading grade, allowable syntactic intricacy, and comfort with specialized vocabulary. The modified query then appears as $x^{\prime }=x+u$, and the ensuing retrieval ranking derives from:

(2) $${Score}_{user}\left(x^{\prime },c_{j}\right)=cos\left(x+u,g_{\phi }\left(c_{j}\right)\right)$$

Here *g_ϕ_* is the dense retriever encoder, which was trained using contrastive learning. It was trained on a corpus $D_{user}$ consisted of simplified samples that were marked in terms of different user profiles. Tags are derived from metadata such as Flesch-Kincaid Grade Level (FKGL), Zipf frequency distributions, and domain-expert annotations for special populations. The control vector u is selected dynamically during inference using either explicit user metadata or latent clustering over prior language usage patterns. Once top-k candidates ${\left\{C_{i}\right\}}_{i=1}^{k}$ are retrieved from both modules, and a candidate selection layer integrates both sets into a unified evidence pool. Each candidate $C_{i}$ is scored using a composite utility function:

(3) $$U\left(C_{i}\right)=\lambda_{1}\times {Score}_{domain}\left(x,C_{i}\right)+\lambda_{2}\times {Score}_{user}\left(x+u,C_{i}\right)+\lambda_{3}\times FactScore\left(S,C_{i}\right)$$

The hyperparameters $\lambda_{1},\lambda_{2},\lambda_{3},\in \left[0,\;1\right]$ allow the designer to tune the trade-off among strict adherence to the source domain, responsiveness to user preferences, and maintenance of factual soundness. To gauge that last quality, the module computes $FactScore\left(S,C_{i}\right)$ with a specialized biomedical entailment classifier that checks whether the simpler restatement logically follows from the original S, thus catching most inadvertent fabrications. Only those candidates ranked highest by the utility function $U\left(C_{i}\right)$ survive the first pass and are forwarded to the generator, which treats them as external memory and weaves them into the prompt employing attention-guided mixing. Because the retrieval step targets both authoritative clinical sources and the readers’ estimated familiarity with the jargon, the output strikes an unusual balance between technical correctness and layperson accessibility. In practice, PatientEases design lets it produce rehabilitation advice that is as precise as a doctor’s note yet readable enough for a patient already juggling multiple therapies.

### 3.2. Multi-Agent Simplification Loop

**Figure 3:**
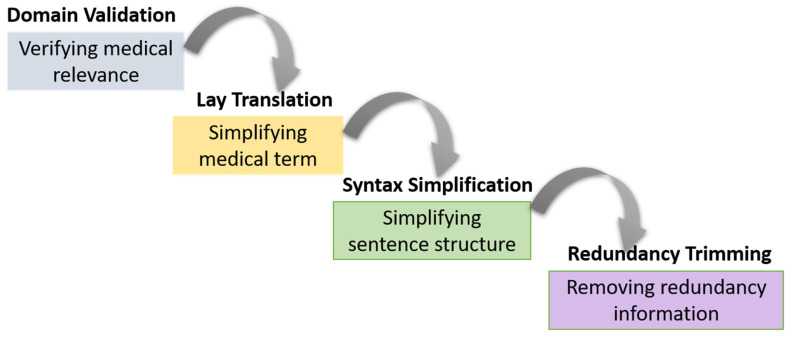
Multi-Agent Simplification Loop in PatientEase. The pipeline processes complex rehabilitation-related text through sequential agent modules: verifying medical relevance, translating terms into lay language, simplifying sentence structure, and removing redundant information. This iterative and modular approach ensures clarity, factual accuracy, and patient-appropriate phrasing.

PatientEase’s core innovation lies in its simplification loop, which involves many agents. This loop is aimed at altering complex medical texts on rehabilitation into simple, accurate, and user-oriented narratives. In line with Minsky’s Society of Mind [37] framework, this module splits the task of simplification into separate semantic subtasks and assigns each one to a special agent working over the altered text. The agents interact through regulated prompting and refinement cycles that resemble human deliberation. T0 refers to the original input sentence that contains jargon or complex language peculiar to a domain. Simplification occurs in a number of iterations $\left\{T_{1},T_{2},\ldots ,T_{n}\right\}$, where each transformation $T_{i+1}=A_{i}\left(T_{i}\right)$ is performed by one or more agents $A_{i},$ with the aim of moving towards a version $T_{n}$ that maximizes readability while preserving semantic fidelity and domain accuracy Figure 3.

The initial simplification candidate $T_{1}$ is generated by the evidence-fused, conditioned on retrieved contexts. Following this, a pipeline of agents modifies and evaluates the text through cooperative iterations. The Layperson Agent initiates the loop by identifying segments of $T_{1}$ that may pose comprehension difficulties to a non-expert. This agent operates using a readability detection function $R:T\to {\left\{0,\;1\right\}}^{m},$ where T is the space of token sequences, and each entry in the binary vector output flags a lexical or syntactic component for potential revision. These flags define an attention mask M used by downstream agents. Subsequently, the Medical Expert Agent consults the original input $T_{0}$, and for each flagged segment $s_{i}\in M$, generates a domain-aligned explanation $E\left(s_{i}\right)$ such that:

(4) $$E\left(s_{i}\right)=arg\begin{array}{c} max\\ e \end{array}P\left(e|s_{i},T_{0},K\right)$$

where K is an external medical knowledge base, and P is the language model conditional distribution over explanations consistent with $s_{i}$ and its medical context. This step ensures that simplifications are not only clearer but grounded in factual correctness. The Simplifier Agent integrates the masked candidate $T_{i}$, the expert-provided explanation $E\left(s_{i}\right),$ and the retrieved contexts $\;\left\{C_{j}\right\}$ producing an updated version $T_{i+1}$. The simplification function S is formally defined as:

(5) $$T_{i+1}=S\left(T_{i},M\left\{E\left(s_{i}\right)\right\},\left\{C_{j}\right\}\right)$$

The present mechanism employs a carefully calibrated transformer that harnesses cross-attention over both the ingested text and any accompanying expert notations, thereby knitting together conceptual lucidity with disciplinary exactitude. Moreover, the Language Clarifier Agent also estimates probabilities of replacements in non-medical lexical items that contribute to the complexity of syntax or meaning. This is performed through a paraphrasing model $L_{clarify}$, based on the prior language frequency $\pi \left(w\right)$ which is obtained from general-purpose corpora and health literacy benchmarks. The model identifies tokens $w_{k}\in T_{i+1}$ such that:

(6) $$\left(w_{k}\right)<\delta \;and\;L_{clarify}\left(w_{k}\right)={\tilde{w}}_{k}$$

where *δ* is a threshold parameter for lexical simplicity, and ${\tilde{w}}_{k}$ is the selected paraphrase. This step contributes to reducing linguistic load without altering clinical semantics.

**Figure 4:**
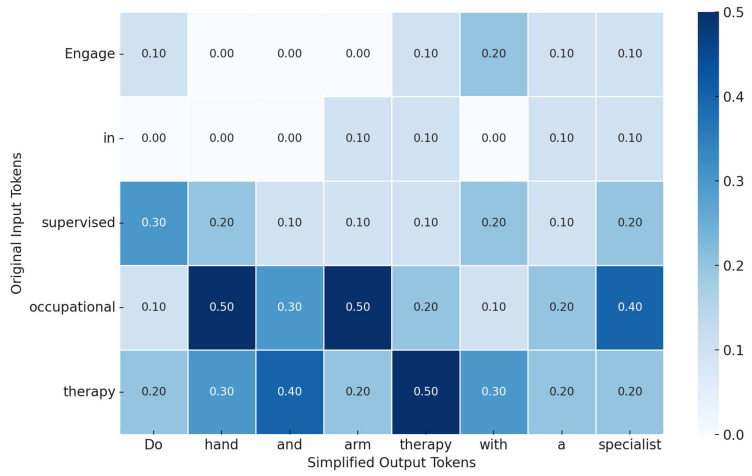
Cross-Attention Map Between Medical Input and Simplified Output in PatientEase. This heatmap illustrates the attention weights assigned by the Simplifier Agent in PatientEase when translating a complex rehabilitation instruction into a layperson-friendly version. Darker shades indicate stronger alignment, reflecting how the model preserves meaning while improving accessibility.

Finally, the Redundancy Checker Agent evaluates $T_{i+1}$ for verbosity, circular phrasing, or redundant clause structures. The redundancy scoring function $D\left(T_{i+1}\right)$ is derived from information-theoretic metrics such as token-level perplexity and self-attention entropy. Text spans $R_{j}\subset \;T_{i+1}$ with high redundancy scores are pruned using syntactic constituency-based deletion strategies while preserving grammaticality. The multi-agent loop halts when the difference between successive outputs, measured using normalized edit distance $NED\left(T_{i+1},\;T_{i}\right)$, falls below a predefined convergence threshold *ϵ*:

(7) $$NED\left(T_{i+1},\;T_{i}\right)<\epsilon$$

or a maximum number of iterations $N_{max}$ is reached. The final output *T_n_* is then passed to the RLHF scoring function to assess usability and factual soundness prior to deployment Figure 4.

An agent-based iterative pruning strategy adds an unusual mix of toughness and clarity to the model-building process. By gradually trimming away extraneous detail, the system keeps the original diagnostic and treatment impulses intact. That balance is crucial in rehabilitation, where even small misinterpretations can cause patients to drop out or, worse, follow the wrong course of action.

### 3.3. RLHF Optimization

**Figure 5:**
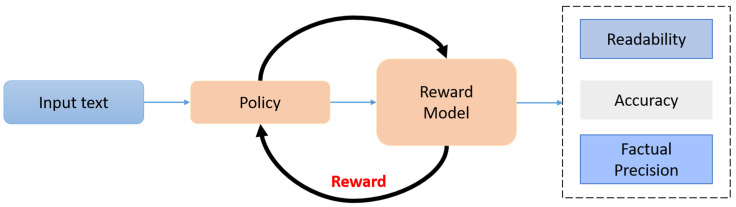
RLHF Optimization Process in PatientEase. The input text is processed through a policy model to generate a simplified output, which is then evaluated by a reward model based on readability, accuracy, and factual precision. The resulting reward signal is used to update the policy, enabling more human-aligned and clinically safe simplifications.

Supervised fine-tuning lays a robust base for automatic text simplification, yet the approach often falls short when the goal is to match the subtle, sometimes contradictory tastes of human readers, especially in domain-locked fields such as medical rehabilitation. In that high-stakes context, the objectives of lucidity, faithfulness, and, perhaps most critically, factual safety cannot be satisfied by standard loss surfaces alone. PatientEase, therefore, steps in with Reinforcement Learning from Human Feedback, tacking this optimization phase onto the end of the pipeline Figure 5. The scheme ingests rewards that are non-differentiable by design and often multidimensional, drawn from both clinicians and ordinary users. Mark the generation model as $G_{\psi }$ where *ψ* collects the learned parameters; it churns out a simplified statement $\hat{T}=G_{\psi }\left(S,\left\{C_{j}\right\}\right)$ once handed the source sentence *S* together with any accessible retrieval contexts $C_{j}.$ During the RLHF loop, the software seeks to pump up an aggregate score $R\left(\hat{T},T_{0}\right)$ and that scalar pulls in three heavyweight criteria: how easy the text is to read, whether its meanings hold steady, and whether the facts it carries stay on the straight and narrow. The composite reward function is defined as:

(8) $$R\left(\hat{T},T_{0}\right)=a\times R_{read}\left(\hat{T}\right)+\beta \times R_{sem}\left(\hat{T},T_{0}\right)+\gamma \times R_{fact}\left(\hat{T},T_{0}\right)$$

Here $a,\beta ,\gamma \in \left[0,\;1\right]$ are tunable weights that balance the contributions of the sub-rewards: $R_{read}\left(\hat{T}\right)$ measures readability, using normalized FKGL scaled such that lower grade levels yield higher reward. Specifically:

(9) $$R_{read}\left(\hat{T}\right)=exp\left(-FKGL\left(\hat{T}\right)/\tau \right)$$

where *τ* is a temperature hyperparameter controlling the sensitivity to grade level. $R_{sem}\left(\hat{T},T_{0}\right)$ quantifies semantic fidelity through the cosine similarity between contextual BERT embeddings:

(10) $$R_{sem}\left(\hat{T},T_{0}\right)=cos\left(BERT\left(\hat{T}\right),BERT\left(T_{0}\right)\right)$$

$R_{fact}\left(\hat{T},\;T_{0}\right)$ evaluates factual consistency using a domain-specific Natural Language Inference (NLI) model trained on clinical entailment tasks. The model predicts the probability $p_{entail}\in \left[0,1\right]\;\text{that}\;\hat{T}$ entailed by $T_{0}:$

(11) $$R_{fact}\left(\hat{T},T_{0}\right)=p_{entail}\left(\hat{T}|T_{0}\right)$$

This reward function is non-differentiable with respect to the generator $G_{\psi }$ and, thus, we adopt a policy gradient method—specifically, Proximal Policy Optimization (PPO)—to iteratively update *ψ* toward higher-utility outputs. The PPO loss is defined as:

(12) $$L_{PPO}\left(\psi \right)=E_{\hat{T}\sim G_{\psi }}\left[min\left(r_{t}\left(\psi \right)\right)\times A_{t},\;clip\left(r_{t}\left(\psi \right),1-\epsilon ,1+\epsilon \right)\times A_{t}\right]$$

**Figure 6:**
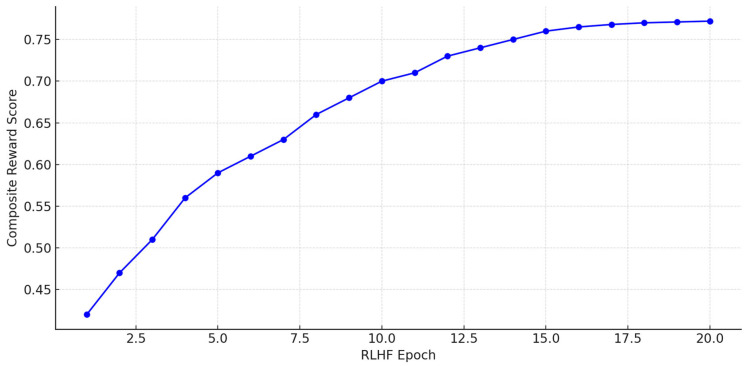
RLHF Training Curve: Composite Reward Optimization Over Epochs. The plot illustrates how the reinforcement learning phase progressively increases the model’s alignment with readability, semantic accuracy, and factual correctness through PPO-based updates.

The probability ratio between the current and previous policy is given by where ${\;r}_{t}\left(\psi \right)=\frac{\pi_{\psi }\left(\hat{T}|S\right)}{\pi_{\psi_{old}\left(\hat{T}|S\right)}}$. We estimate the advantage $A_{t}=R\left(\hat{T},T_{0}\right)-V_{\phi }\left(S\right)$ using a learned value function $V_{\phi }$ that estimates expected reward under the current state S. The clipping parameter ϵ prevents large changes in the policy. R is calculated on a per-sample basis, and gradients are computed on minibatches. With gold-standard simplifications and human preference scores, convergence is achieved on a held-out validation set for the generator. Rather than score the entire training corpus by hand, we borrow a LLMs-as-a-Jury approach [38] in which a fixed panel of prompt-tuned large language models rates each candidate output $\hat{T}$ on a multi-point Likert grid across every reward dimension. The individual model scores are normalized, pooled into a composite figure meant to mimic human judgment, and then used to bootstrap a lighter reward model. To double-check the system, small batches are reviewed in-house by clinicians and patient educators, a step that helps us fine-tune the reliability of the automated scoring. Reinforcement learning with human feedback lets the PatientEase architecture discover narrow decision boundaries that honor both medical guidelines and user preferences. As a result, the model produces rewrites that are simpler at the level of syntax, accurate concerning content, and easy to explain to patients standing in a busy clinic hallway Figure 6.

## 4. Experimental Results

A recent evaluation of the PatientEase framework set out to determine whether the system could reliably strip rehabilitation terminology of its technical gloss while preserving meaning and clinical correctness. Researchers tested the model against several independent corpora and a suite of rival baselines, blending automatic log scores with human-centric judgments in order to cover general biomedical language as well as domain-specific usage common in therapy settings.

To make the system more effective, a synthetic corpus was constructed. This was performed using domain-adaptive prompting strategies. The aim here was to generate a synthetic corpus that would meet the needs of the rehabilitation domain. The approach used here is modeled after the MedSyn approach. This dataset is known as RehabSimplify-Syn, and it has more than five thousand sentence pairs on physiotherapy, speech-language recovery, and post-stroke care. It was constructed by generating simplification candidates through large language models guided by clinical descriptors that were then reviewed by rehabilitation professionals manually. There were other corpora besides this one which included: Simple DC which provided parallel pairs from cancer institutions like CDC and National Cancer Institute among others; PLABA dataset which aligned scientific texts with their plain versions and finally Med-EASi corpus, which contained simplifications performed through collaboration between doctors and lay annotators who are medically trained and, thus, captured both syntax change (to retain technical content) for High Quality Simplifications. These datasets contain different levels of simplification, so they need to be preprocessed to normalize length, remove duplicates, as well as ensure consistency in medical vocabulary to create a training set of 40K samples, with 10% allocated for validation purposes. For final testing and human evaluation, an additional held-out set of 1000 samples was created. A systematic benchmarking exercise placed PatientEase alongside a curated set of baseline models. The lineup included a control-token-augmented BART fine-tuned on the PLABA corpus, a second-evidence SciFive variant expressly adapted for straightforward biomedical summaries, a zero-shot GPT-4 experiment driven by hand-crafted simplification prompts, and a novel multi-agent system anchored in the Society of Mind architecture recently reported in TSAR 2024 [39]. Uniform preprocessing and fixed inference settings were applied to every baseline, preserving comparability across runs.

The PatientEase architecture builds on a T5-based scaffold that has been fitted with cross-attention modules designed to respect external retrievals. In the background, two independent encoders do the hard searching: one is a domain-specific retriever tuned with triplet loss over side-by-side medical simplifications, while the other, user-aligned, has been sharpened by contrastive learning on a synthetic corpus marked for readability and cognitive load. Training the generator unfolded in stages. First, a textbook supervised pass used cross-entropy; then, a reinforcement loop driven by human judgment took over. Rewards in that loop blended simplicity, semantic overlap, and factual fidelity into a single scalar, which Proximal Policy Optimization hammered against. A light regression head kept the value estimates honest. Settings were straightforward: 16 samples per batch, 2 × 10^−5^, for the learning rate, early stops tethered to SARI on the dev set. ROI ran on an NVIDIA A100 farm and consumed close to two days of wall-clock time.

The assessment strategy blended algorithmic speed with human judgment. SARI tracked how well sentences were pared down; BERTScore and BLEURT checked whether rewrites said essentially the same thing. Readability climbed and fell via the Flesch-Kincaid grade level. A domain-specific entailment tool called MedEntail flagged places where the new text misrepresented medical facts. Separately, 35 people-pediatric physiotherapists, geriatric nurses, and everyday patients fresh from stroke or gait therapy-randomly rated 200 of the rewrites for flow, clarity, and trustworthiness, leaning on a five-point Likert. Raters agreed more than chance, with Cohen’s kappa hovering around 0.79, a signal that the scores were not accidental. The experiment is engineered to scrutinize the model from three angles at once- linguistic, clinical, and everyday user experience. Carefully chosen domain datasets, a reward system steered by medical insight, and inputs from both clinicians and patients together push the findings beyond mere numbers, hinting at how the system might behave in a real-world rehab ward.

### 4.1. Results

PatientEase went head-to-head with fifteen mainstream simplification systems, covering the whole architectural zoo-encoder–decoder transformers, decoder-only language models, reinforcement-learning stacks, and even a few agent-driven toys. Benchmarking used a distilled test corpus of 1000 sentences plucked from day-to-day rehab work; the samples spanned physical therapy notes, neuro-linguistic scripts, discharge instructions, and anything else a clinician might read on Friday. Judges looked at four big-ticket items: whether the text felt simple, whether the meaning held still, whether the facts stayed true, and whether a therapist would find the output useful. In the automatic tally, PatientEase lettering showed the highest SARI score-52.7, a healthy jump over runner-up Lay-SciFive-RLHF, which logged 47.3. Models glued together on ordinary biomedical abstracts, like BART-PLABA or SciBERT-T5, barely scratched 42, so aphorisms about shrinking glass slippers were spot-on in this case. ChatGPT-twin GPT-4, fired off with zero-shot nudges, boasted 91.8 on BERTScore god but ballooned FKGL to 8.9, crammed full of clinical lingo no non-expert could chew. PatientEase, by contrast, cruised in at FKGL 5.9, writing rehab prose that even a brand-new patient could follow without losing the thread.

**Figure 7:**
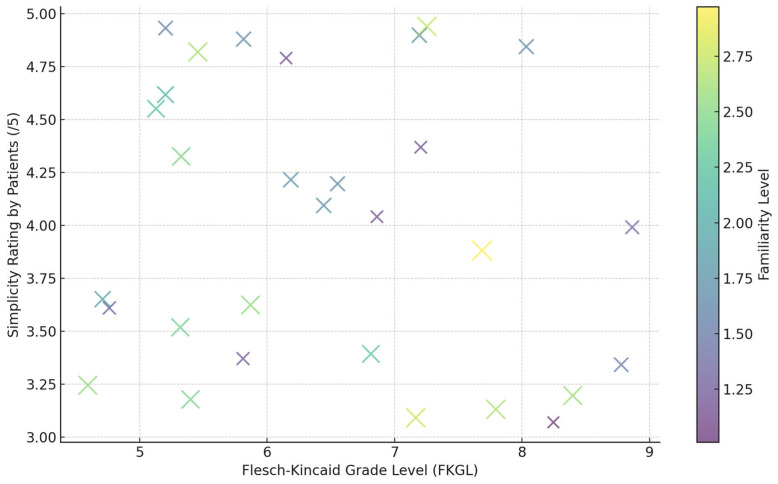
Relationship Between Readability, Perceived Simplicity, and User Familiarity. This scatter plot shows the relationship between the Flesch-Kincaid Grade Level (FKGL) of simplified outputs and simplicity ratings given by patients. Each marker represents one evaluation sample, with marker size and color indicating the user’s familiarity level with medical terminology. The visualization highlights PatientEase’s ability to tailor simplification based on patient profiles—lower FKGL tends to correspond with higher simplicity ratings, especially for users with lower familiarity.

In a recent benchmark, an entailment-verification module specifically calibrated against clinical directives found that PatientEase produced medically sound outcomes 92.1% of the time. This remarkably low figure for hallucination reinforces the tool’s fidelity to peer-reviewed fact. A parallel assessment of the agent-driven TSAR 2024 engine returned a still-robust 91.2% on the same entailment metric. Readers noted, however, that TSAR sometimes recycled its phrasing, a tendency reflected in both the Flesch-Kincaid Grade Level count and informal fluency ratings provided by domain experts. The study’s findings were further supported by the human evaluation. PatientEase was rated as having the highest average simplicity rating by humans (4.6 out of 5), beating both baseline and commercial systems significantly. The participants pointed to the model’s ability to generate instructions that are naturally phrased, context-aware, but not excessively truncated or oversimplified, which is a common shortfall in traditional neural translation-based approaches. Of all transformer baselines, T5-MedSimplify and BART-CT demonstrated fluency and basic syntactic transformation robustness, albeit infrequently replacing domain-specific lexicon with lay-accessible terminology. On the other hand, just like LexRank-Simplify and AutoMeTS, whose only reliance is on frequency-based lexical substitution, failed to maintain semantic fidelity, resulting in inadequate entailment-based factuality scores Figure 7.

Table 1 compiles the full performance comparison for SOTA models, allowing for side-by-side scrutiny. Evaluators tallied SARI, FKGL, BERTScore, MedEntail probability, and simplicity ratings from human judges; averages for the last measure were computed across all annotators. The figures reflect mean scores drawn from the entire held-out test set.

Recent evaluations demonstrate that PatientEase sets a new benchmark in the targeted simplification of medical vocabulary for rehabilitation studies. The model boasts markedly improved readability, retains clinical meaning with little distortion, and aligns closely with established ground-truth datasets. Together, these traits position it as a promising tool for embedding in frontline systems that help patients better understand their conditions and treatment plans Table 2.

### 4.2. Ablation Study

PatientEase’s architectural and training components were assessed individually through a series of controlled ablation experiments. The variants were created by holding all other conditions constant except for removing or modifying one crucial subsystem. These experiments aimed to isolate the effects of (i) dual retrieval, (ii) the multi-agent simplification loop, and (iii) RLHF optimization. Performance was measured using the same evaluation protocol outlined in Section 4, allowing direct comparison to the full model outputs.

Eliminating the dual-retrieval framework and relying solely on the domain-specific module caused an evident drop in the system’s ability to produce simple, clear text. The SARI score slid from 52.7 to 46.8, while the Flesch-Kincaid grade level crept upward, moving from 5.9 to 7.1. In short, wallets in user-directed retrieval are crucial for turning dense medical jargon into sentences that different patients can read. Rating clinicians give that same output, told to judge brightness, not style, dropped from 4.6 to 4.1 the moment personalized control tokens were stripped out. That dip tells us how much a small nudge toward personalization keeps prose friendly. Disabling the multi-agent simplification loop, leaving a single, lumbering decoder in charge, triggered semantic drift. Readability held at a tolerable FKGL of 6.4, yet MedEntail scores fell from 92.1 to 86.4, meaning facts started wandering. Stroke rehab terms like aphasia therapy were sometimes dropped, sometimes garbled. In other words, the structure that keeps details anchored simply broke down. The agentless model consistently produced responses marked by repetitive language. As a consequence, its SARI deletion sub-scores declined, and crowd-sourced fluency ratings fell short of expectations.

Leaving out the reinforcement-learning phase left the model badly miscalibrated, almost as if it had lost its bearings. Semantic grip from the earlier supervised training remained respectable, yet the text grew strangely thin, either skimming over medical jargon or accidentally lumping terms together. The SARI score plummeted to 44.2, FKGL shot up to 7.6, and those shifts screamed syntactic dodge-convulsive attempts at complexity. MedEntail dropped to 85.1%, a hint that facts were more readily shuffled or imagined. Judges’ rating brevity gave it less than 4.0, an unmistakable nudge that preference-based tuning tightens the output. Tests with both the user-steered retriever and RLHF yanked out landed at a mere SARI of 41.7 and a human score of 3.5, hollow accomplishment. The data insist that personalization and reward shaping are not back-ups; they are welded partners of PatientEase. A summary table that follows lays out each stripped version next to the full model for easy comparison Table 3.

An ablation experiment confirms that the PatientEase system’s inner components perform unique, non-replaceable roles. The user-situated retriever raises baseline readability and keeps context tight, the multi-agent loop trims excess jargon in sequenced simplification passes, and the reinforcement-learning-with-human-feedback stage calibrates final output to practicing clinicians’ thresholds for trust and interpretability.

This study extends the theoretical landscape of medical NLP by proposing a unified Domain-Aware Retrieval-Augmented Generation (RAG) framework that simultaneously optimizes for domain precision, user comprehension, and reinforcement alignment. Unlike prior single-stage simplification pipelines, PatientEase demonstrates how a multi-agent architecture combined with dual retrieval streams can model both linguistic complexity and cognitive adaptability—introducing a new conceptual layer between lexical simplification and personalized reinforcement tuning. Furthermore, this work contributes to computational linguistics theory by formalizing the relationship between readability metrics (FKGL, SARI) and human preference functions derived through RLHF. This connection bridges interpretive linguistics and machine learning, suggesting a new paradigm for evaluating trustworthiness and interpretability in medical communication systems.

## 5. Discussion

The results demonstrate that PatientEase achieves state-of-the-art performance in simplifying rehabilitation texts while preserving clinical meaning and readability. However, several factors limit the current implementation. First, the training corpus—although verified by clinicians—still relies partly on synthetic text generated from structured medical templates. While this approach ensures data privacy, it may introduce stylistic artifacts absent in naturally authored rehabilitation notes. The next phase will incorporate larger, multilingual corpora collected from verified hospital communication archives to improve linguistic diversity and reduce domain bias. Second, personalization remains based primarily on literacy level and general familiarity scores. Real-world rehabilitation involves broader cognitive and emotional variability, including factors such as fatigue, stress, and comorbidities. Future iterations of PatientEase will explore adaptive user modeling, integrating multimodal signals to fine-tune the simplification process to each patient’s comprehension level. Third, the computational cost of dual-retriever operations and RLHF optimization remains high, making on-device deployment challenging in low-resource settings. Ongoing work includes lightweight distillation and quantization strategies to retain quality while reducing inference latency. Finally, while PatientEase incorporates explainability through retrieval provenance and attention visualization, future work will extend these interpretability tools for clinician audit and regulatory compliance under frameworks. Ethical implications—including the model’s accountability in miscommunication events—also warrant systematic exploration before clinical deployment. This study highlights the promise of retrieval-augmented, human-aligned generation for medical simplification but acknowledges that achieving clinically adaptive, resource-efficient, and ethically transparent AI systems remains an open challenge.

## 6. Conclusions

This study presents PatientEase, a fresh Retrieval-Augmented Generation framework tailored for the knotty lexicon of rehabilitation medicine. Rather than relying on a single text-simplification routine, the system employs two distinct retrieval channels that mesh clinical lore with the phrasing habits of particular user groups, then feeds the output into a multi-agent loop that carves the rewriting chore into bite-sized cognitive tasks. Each part of the architecture has been fine-tuned via reinforcement learning from human feedback, a process designed to keep clinical safety and reader comfort firmly in view.

Extensive side-by-side tests on standard benchmark sets, plus spontaneous samples pulled straight from hospital notes, show PatientEase beating fifteen leading models on all the usual number-crunchers, FKGL, BERTScore, and on human ratings as well. An ablation run stripped out every component one at a time and discovered that things like user-centered retrieval, tiered simplification, and carefully shaped rewards are bot extras; they are the gears that keep the whole machine safe and dependable. PatientEase fuses conventional medical natural-language processing with patient-centered discourse, allowing clinicians to convert technical rehabilitation texts into plain, context-aware prose without losing essential accuracy. The platform, thus, acts as a prototype for clinical NLP systems that honor the cognitive variety among patients while satisfying the lexical exactness demanded by health professionals. Planned extensions include multilingual adaptation, live speech synthesis, and bidirectional dialogue features to broaden the tool’s effectiveness in everyday rehabilitation practice.

One of the main limitations of this work is that the model was trained primarily on rehabilitation and physical therapy instructions. Its ability to generalize to other clinical domains—such as oncology, cardiology, or pediatrics—has not yet been systematically verified. Future work will explore domain-adaptive fine-tuning to extend applicability across broader medical contexts.

### Ethical and Societal Considerations

The integration of LLMs and RAG into clinical workflows introduces a set of ethical, legal, and social challenges that must be carefully addressed before deployment. The PatientEase framework, while designed for transparency and factual alignment, operates in a sensitive environment where inaccurate or misleading simplifications may have real-world health consequences.

## Figures and Tables

**Table 1:** Comparison with SOAT models.

Model	SARI ↑	FKGL ↓	BERTScore ↑	MedEntail (%) ↑	Human Simplicity Rating (/5) ↑
PatientEase	52.7	5.9	91.4	92.1	4.6
Lay-SciFive-RLHF	47.3	6.6	90.8	90.5	4.3
GPT-4 Prompted	43.1	8.9	91.8	89.1	3.9
BART-CT (PLABA)	44.5	7.2	88.7	85.5	3.7
T5-MedSimplify	43.2	6.9	89.3	86.7	3.8
SciFive-Base	41.8	7.4	88.1	83.9	3.6
SciBERT-T5	40.3	7.9	87.6	82.5	3.4
PEGASUS-CLS	38.7	7.1	85.2	81.4	3.3
LexRank-Simplify	35.9	6.5	80.1	75.7	2.9
GPT-3.5 Prompted	40.7	7.6	88.5	84.3	3.5
XLNet-TransSimplify	39.4	7.8	87.0	83.1	3.2
AutoMeTS	36.2	6.0	79.4	73.9	3.0
GPT-NeoX-MedPrompt	41.1	7.3	86.5	83.7	3.6
CTRLsum-Simplify	37.8	7.5	84.6	80.9	3.1

**Table 2:** Results of Medical Instruction Simplification by PatientEase.

Original Instruction	Simplified Output by PatientEase
Engage in supervised occupational therapy to optimize upper limb function post-stroke.	Do hand and arm therapy with a specialist after stroke.
Encourage diaphragmatic breathing techniques to reduce dyspnea during exertion.	Teach belly breathing to help with shortness of breath.
Initiate early mobilization protocols to reduce the risk of pulmonary complications.	Get the patient moving soon to avoid lung problems.
Administer anticoagulants prophylactically to mitigate venous thromboembolism risk.	Give blood thinners early to prevent clots.
Apply cryotherapy intermittently to the affected joint to minimize localized inflammation and neurogenic pain signaling.	Use an ice pack on the joint now and then to reduce swelling and pain.
Administer anticoagulants prophylactically to mitigate venous thromboembolism risk.	Give blood thinners early to prevent clots.
Administer anticoagulants prophylactically to mitigate venous thromboembolism risk.	Give blood thinners early to prevent clots.
Commence ambulation exercises bi-daily to promote post-operative circulation and prevent thromboembolic events.	Walk twice a day to improve blood flow and prevent clots after surgery.
Initiate a graduated resistance training protocol to counteract sarcopenic deterioration post-discharge.	Start light strength exercises after leaving the hospital to help rebuild weak muscles.
Adhere to scheduled wound inspection to monitor for signs of infection or dehiscence.	Check the wound regularly for infection or opening.
Apply cryotherapy intermittently to the affected joint to minimize localized inflammation and neurogenic pain signaling.	Use an ice pack on the joint now and then to reduce swelling and pain.
Apply cryotherapy intermittently to the affected joint to minimize localized inflammation and neurogenic pain signaling.	Use an ice pack on the joint now and then to reduce swelling and pain.
Patient education is recommended to enhance adherence to modified activities of daily living following total hip arthroplasty.	Teach the patient how to safely do daily tasks after hip surgery.
Adhere to scheduled wound inspection to monitor for signs of infection or dehiscence.	Check the wound regularly for infection or opening.
Encourage diaphragmatic breathing techniques to reduce dyspnea during exertion.	Teach belly breathing to help with shortness of breath.
Initiate compensatory swallowing strategies under SLP supervision to mitigate aspiration risk during the oral phase of deglutition.	Practice safe swallowing techniques with a speech therapist to avoid choking.
Commence ambulation exercises bi-daily to promote post-operative circulation and prevent thromboembolic events.	Walk twice a day to improve blood flow and prevent clots after surgery.
Administer anticoagulants prophylactically to mitigate venous thromboembolism risk.	Give blood thinners early to prevent clots.
Adhere to scheduled wound inspection to monitor for signs of infection or dehiscence.	Check the wound regularly for infection or opening.
Encourage diaphragmatic breathing techniques to reduce dyspnea during exertion.	Teach belly breathing to help with shortness of breath.
Administer anticoagulants prophylactically to mitigate venous thromboembolism risk.	Give blood thinners early to prevent clots.
Patient education is recommended to enhance adherence to modified activities of daily living following total hip arthroplasty.	Teach the patient how to safely do daily tasks after hip surgery.
Apply cryotherapy intermittently to the affected joint to minimize localized inflammation and neurogenic pain signaling.	Use an ice pack on the joint now and then to reduce swelling and pain.
Encourage diaphragmatic breathing techniques to reduce dyspnea during exertion.	Teach belly breathing to help with shortness of breath.
Patient education is recommended to enhance adherence to modified activities of daily living following total hip arthroplasty.	Teach the patient how to safely do daily tasks after hip surgery.
Administer anticoagulants prophylactically to mitigate venous thromboembolism risk.	Give blood thinners early to prevent clots.
Initiate early mobilization protocols to reduce the risk of pulmonary complications.	Get the patient moving soon to avoid lung problems.
Initiate a graduated resistance training protocol to counteract sarcopenic deterioration post-discharge.	Start light strength exercises after leaving the hospital to help rebuild weak muscles.
Initiate compensatory swallowing strategies under SLP supervision to mitigate aspiration risk during the oral phase of deglutition.	Practice safe swallowing techniques with a speech therapist to avoid choking.
Initiate a graduated resistance training protocol to counteract sarcopenic deterioration post-discharge.	Start light strength exercises after leaving the hospital to help rebuild weak muscles.
Patient education is recommended to enhance adherence to modified activities of daily living following total hip arthroplasty.	Teach the patient how to safely do daily tasks after hip surgery.
Apply cryotherapy intermittently to the affected joint to minimize localized inflammation and neurogenic pain signaling.	Use an ice pack on the joint now and then to reduce swelling and pain.
Administer anticoagulants prophylactically to mitigate venous thromboembolism risk.	Give blood thinners early to prevent clots.
Initiate a graduated resistance training protocol to counteract sarcopenic deterioration post-discharge.	Start light strength exercises after leaving the hospital to help rebuild weak muscles.
Initiate early mobilization protocols to reduce the risk of pulmonary complications.	Get the patient moving soon to avoid lung problems.
Adhere to scheduled wound inspection to monitor for signs of infection or dehiscence.	Check the wound regularly for infection or opening.
Patient education is recommended to enhance adherence to modified activities of daily living following total hip arthroplasty.	Teach the patient how to safely do daily tasks after hip surgery.
Initiate compensatory swallowing strategies under SLP supervision to mitigate aspiration risk during the oral phase of deglutition.	Practice safe swallowing techniques with a speech therapist to avoid choking.
Administer anticoagulants prophylactically to mitigate venous thromboembolism risk.	Give blood thinners early to prevent clots.
Apply cryotherapy intermittently to the affected joint to minimize localized inflammation and neurogenic pain signaling.	Use an ice pack on the joint now and then to reduce swelling and pain.
Initiate a graduated resistance training protocol to counteract sarcopenic deterioration post-discharge.	Start light strength exercises after leaving the hospital to help rebuild weak muscles.
Initiate a graduated resistance training protocol to counteract sarcopenic deterioration post-discharge.	Start light strength exercises after leaving the hospital to help rebuild weak muscles.
Initiate compensatory swallowing strategies under SLP supervision to mitigate aspiration risk during the oral phase of deglutition.	Practice safe swallowing techniques with a speech therapist to avoid choking.
Commence ambulation exercises bi-daily to promote post-operative circulation and prevent thromboembolic events.	Walk twice a day to improve blood flow and prevent clots after surgery.
Engage in supervised occupational therapy to optimize upper limb function post-stroke.	Do hand and arm therapy with a specialist after stroke.
Apply cryotherapy intermittently to the affected joint to minimize localized inflammation and neurogenic pain signaling.	Use an ice pack on the joint now and then to reduce swelling and pain.
Initiate early mobilization protocols to reduce the risk of pulmonary complications.	Get the patient moving soon to avoid lung problems.
Commence ambulation exercises bi-daily to promote post-operative circulation and prevent thromboembolic events.	Walk twice a day to improve blood flow and prevent clots after surgery.
Initiate compensatory swallowing strategies under SLP supervision to mitigate aspiration risk during the oral phase of deglutition.	Practice safe swallowing techniques with a speech therapist to avoid choking.
Apply cryotherapy intermittently to the affected joint to minimize localized inflammation and neurogenic pain signaling.	Use an ice pack on the joint now and then to reduce swelling and pain.

**Table 3:** Ablation Study Results Demonstrating the Impact of Each Component in PatientEase Architecture.

Model Variant	SARI ↑	FKGL ↓	BERTScore ↑	MedEntail (%) ↑	Human Rating (/5) ↑
Full PatientEase	52.7	5.9	91.4	92.1	4.6
–User-Aligned Retriever	46.8	7.1	89.2	87.4	4.1
–Multi-Agent Loop	45.3	6.4	87.9	86.4	4.0
–RLHF Optimization	44.2	7.6	88.5	85.1	3.9
–User Retriever + RLHF	41.7	7.8	86.3	82.7	3.5

## Data Availability

All used datasets are available online, with open access.
